# Health data privacy through homomorphic encryption and distributed ledger computing: an ethical-legal qualitative expert assessment study

**DOI:** 10.1186/s12910-022-00852-2

**Published:** 2022-12-01

**Authors:** James Scheibner, Marcello Ienca, Effy Vayena

**Affiliations:** 1 Health Ethics and Policy Laboratory, Department of Health Sciences and Technology (D-HEST), ETH Zürich, Zurich, Switzerland; 2 College of Business, Government and Law, Flinders University, Adelaide, Australia; 3 College of Humanities, EPFL, Lausanne, Switzerland; 4 Department of Health Sciences and Technology, ETH Zürich, Zurich, Switzerland

**Keywords:** Data protection, Privacy preserving technologies, Qualitative research, Vignettes, Interviews, Distributed ledger technology, Homomorphic encryption

## Abstract

**Background:**

Increasingly, hospitals and research institutes are developing technical solutions for sharing patient data in a privacy preserving manner. Two of these technical solutions are homomorphic encryption and distributed ledger technology. Homomorphic encryption allows computations to be performed on data without this data ever being decrypted. Therefore, homomorphic encryption represents a potential solution for conducting feasibility studies on cohorts of sensitive patient data stored in distributed locations. Distributed ledger technology provides a permanent record on all transfers and processing of patient data, allowing data custodians to audit access. A significant portion of the current literature has examined how these technologies might comply with data protection and research ethics frameworks. In the Swiss context, these instruments include the Federal Act on Data Protection and the Human Research Act. There are also institutional frameworks that govern the processing of health related and genetic data at different universities and hospitals. Given Switzerland’s geographical proximity to European Union (EU) member states, the General Data Protection Regulation (GDPR) may impose additional obligations.

**Methods:**

To conduct this assessment, we carried out a series of qualitative interviews with key stakeholders at Swiss hospitals and research institutions. These included legal and clinical data management staff, as well as clinical and research ethics experts. These interviews were carried out with two series of vignettes that focused on data discovery using homomorphic encryption and data erasure from a distributed ledger platform.

**Results:**

For our first set of vignettes, interviewees were prepared to allow data discovery requests if patients had provided general consent or ethics committee approval, depending on the types of data made available. Our interviewees highlighted the importance of protecting against the risk of reidentification given different types of data. For our second set, there was disagreement amongst interviewees on whether they would delete patient data locally, or delete data linked to a ledger with cryptographic hashes. Our interviewees were also willing to delete data locally or on the ledger, subject to local legislation.

**Conclusion:**

Our findings can help guide the deployment of these technologies, as well as determine ethics and legal requirements for such technologies.

**Supplementary Information:**

The online version contains supplementary material available at 10.1186/s12910-022-00852-2.

## Introduction

Advanced technological solutions are increasingly used to resolve privacy and security challenges with clinical and research data sharing [1, 2]. The legal assessment of these technologies so far has focussed on compliance. Significant attention has been paid to the modernised General Data Protection Regulation (GDPR) of the European Union (EU) and its impacts. In particular, the GDPR restricts the use and transfer of sensitive personal data, including genetic, medical and health related data. Further, under the GDPR, data custodians and controllers must take steps to ensure the auditability of personal data, including patient data. Specifically, the GDPR’s provisions guaranteeing the right to access information about processing (particularly automated processing) allow data subjects to monitor the use of their data [3]. These rights exist alongside requirements for data custodians and controllers to keep records of how they have processed personal data [4]. The GDPR also introduces a right of erasure, which allows an individual to request that a particular data controller or data custodian delete their data.

At the same time, the increased use of big data techniques in health research and personalised medicine has led to a surge in hospitals and healthcare institutions collecting data [1]. Therefore, guaranteeing patient privacy, particularly for data shared between hospitals and healthcare institutions, represents a significant technical and organisational challenge. The relative approach to determining anonymisation under the GDPR means that whether data is anonymised depends on both the data and the environment in which it is shared. Accordingly, at present it is unclear whether the GDPR permits general or “broad” consent for a research project [5]. A further issue concerns how patient data might be used for research. On the one hand, patients are broadly supportive of their data being used for research purposes or for improving the quality of healthcare. On the other hand, patients have concerns about data privacy, and their data being misused or handled incorrectly [6].

In response to these challenges, several technological solutions have emerged to aid compliance with data protection legislation [7]. Two examples of these with differing objectives are homomorphic encryption (HE) and distributed ledger technology (DLT). HE can allow single data custodians to share aggregated results without the need to share the data used to answer that query [8, 9]. For example, HE can be particularly useful for performing queries on data which must remain confidential, such as trade secrets [10]. In addition, as we discuss in this paper, HE can be useful for researchers who wish to conduct data discovery or feasibility studies on patient records [11]. A feasibility study is a piece of research conducted before a main research project. The purpose of a feasibility study is to determine whether it is possible to conduct a larger structured research project. Feasibility studies can be used to assess the number of patients required for a main study, as well as response rates and strategies to improve participation [12]. A challenge with conducting feasibility studies is that datasets may be held by separate data custodians at multiple locations (such as multiple hospitals). To conduct a feasibility study, the data custodian at each location would need to guarantee data security before transferring the data. Although necessary, this process can be time consuming and due to the need for bespoke governance arrangements [13]. However, HE can allow for data discovery queries to be performed on patient records without the need for that data to be transferred [11]. With adequate organisational controls, the lack of transfer of data using HE could satisfy the GDPR’s definition of anonymised data and state of the art encryption measures [2, 7].

By contrast, DLT is not designed to guarantee privacy, but to increase transparency and trust. DLT attempts to achieve this objective by offering each agent in a processing network a copy of a chain of content. This chain of content, known as the ledger, is read only, and all access to the content on it is timestamped. Accordingly, this ledger provides all agents with a record of when access to data occurs [14]. The ledger uses a cryptographic “proof of work” algorithm before new records can be added to prevent tampering [15]. Perhaps the most famous use case for DLT is Blockchain, which is designed to record agents transacting with digital assets [16]. However, proof of work algorithms were initially employed to certify the authenticity of emails and block spam messages [17]. Further, a similar algorithm to that employed in many Blockchain implementations was initially used to record the order in which digital documents were created [14, 18]. These examples demonstrate that there may be possible uses for DLT outside currency implementations [18]. Accordingly, some scholars have proposed using DLT style implementations to create an auditable record of access to patient data [19, 20]. Proponents of this approach argue that access to an auditable record could help increase patient trust in the security of their data [21, 22]. Further, DLT could be coupled with advanced privacy enhancing technologies to enable auditing for feasibility studies and ensure that only authorised entities can access patient records [20]. Nevertheless, there are ongoing questions as to the degree to which HE and DLT can be used to store and process personal data whilst remaining GDPR compliant [7, 23]. In particular, the read only nature of the ledger underpinning DLT might conflict with the right of erasure contained in the GDPR. The relationship between data protection law and these novel technologies is further complicated when considering data transfer outside the EU [24, 25]. Beyond these legal considerations, there are deeper normative issues regarding the relationship between individual privacy and the benefits flowing from information exchange. These issues are particularly pronounced when dealing with healthcare, medical or biometric data, where patients are forced into an increasingly active role in how their data is used [26].

Accordingly, the purpose of this paper is to assess the degree to which novel privacy enhancing technologies can assist key data custodian stakeholders in complying with regulations. For this paper, we will focus on the case study of Switzerland. Switzerland is not an EU member state and is therefore not required to implement the GDPR into its national data protection law (the *Datenschutzgesetz*, or Federal Act on Data Protection (FADP)). However, because of Switzerland’s regional proximity to other EU member states, there is significant data transfer between Swiss and EU data custodians [27]. Accordingly, the ongoing transfer of data between Switzerland and the EU requires the FADP to offer adequate protection as assessed under Article 45 of the GDPR [28, 29]. Because the FADP was last updated in 1992, the Swiss Federal Parliament in September 2020 passed a draft version of the updated FADP. Estimated to come into effect in 2022, this FADP is designed to achieves congruence with the GDPR [30]. Further, Switzerland also has separate legislation concerning the processing of health related and human data for medical research, the Human Research Act (HRA) and the Human Research Ordinance (HRO). These instruments impose additional obligations beyond the FADP for the processing of health-related data for scientific research purposes [29, 31]. Crucially, the Human Research Act permits the reuse of health-related data for future secondary research subject to consent and ethics approval. The Human Research Act also creates a separate regime of genetic exceptionalism for non-genetic and genetic data [32]. Specifically, anonymised genetic data requires the patient “not to object” to secondary use, whilst anonymised non-genetic data can be transferred without consent. Under the HRA, research with coded health-related data can be conducted with general consent, whilst coded genetic data requires consent for a specific research project [1]. Recent studies indicate that whilst most health research projects in Switzerland use coded data [33]. This definition is considered analogous to pseudonymised data under the GDPR [1, 34]. Finally, Switzerland is a federated country, with different cantonal legislation for data protection and health related data. This federated system has the potential to undermine nationwide strategies for interoperable data sharing. Therefore, the Swiss Personalised Health Network (SPHN) was established to encourage interoperable patient data sharing. In addition to technical infrastructure, the SPHN has provided a governance framework to standardise the ethical processing of health-related data by different hospitals [31].

In this paper we describe a series of five vignettes designed to test how key stakeholders perceive using HE and DLT for healthcare data management. The first three vignettes focus on governing data transfer with several different types of datasets using HE. These include both genetic and non-genetic data, to capture the genetic exceptionalism under the Swiss regulatory framework. The latter two vignettes focus on erasure requests for records of data stored using DLT. We gave these vignettes to several key stakeholders in Swiss hospitals, healthcare institutions and research institutions to answer. We then transcribed and coded these answers and offer recommendations in this paper regarding the use of these technologies for healthcare management. Our paper is the product of an ethico-legal assessment of HE and DLT conducted as part of the Data Protection and Personalised Health (DPPH) project. The DPPH project is an SPHN-Personalized Health and Related Technologies (PHRT) driver project designed to assess the use of these technologies for multisite data sharing. Specifically, the platform MedCo, which is the main technical output from the DPPH project, uses HE for data feasibility requests on datasets stored locally at Swiss hospitals [11]. Access to this data will be monitored using decentralised ledger technology that will guarantee auditability. Therefore, the purpose of this study is to empirically assess the degree to which the technologies above can help bridge the gap between these multiple layers of regulation.

## Methods

To explore the impact of these technologies on regulation governing health data sharing, we conducted an interview study with expert participants in the Swiss health data sharing landscape. Our interviewees were drawn from legal practitioners, ethics practitioners and clinical data managers working at Swiss hospitals, research institutes and governance centres. We used vignette studies, with two sets of vignettes for each technology we tested. For this part of our project, we developed an interview study protocol with vignettes, describing two parts dedicated to the two technologies described above. Vignettes are a useful tool for both qualitative and quantitative research, as well as research with small and large samples. In qualitative research, they allow for both structured data and comparison of responses [35, 36]. Further, vignettes can provoke interviewees to explain the reasons for their responses more comprehensively than open ended questions [37]. Accordingly, for this study vignettes were chosen because we not only wanted to assess perspectives on specific technologies but also how various stakeholders would apply these principles in practice [38]. Our findings can help assess the degree to which these technologies can help bridge the gap between different layers of the regulatory framework described above.

Part A of our vignette contained three scenarios concerning a data discovery or feasibility request on patient data. These scenarios were designed using example data discovery protocols from the DPPH project [11]. The first scenario pertained to a data discovery request on non-genetic patient data to build a cohort of patients who had received an anti-diabetic drug. The second scenario pertained to a data discovery request on genetic patient data to build a cohort of patients with biomarkers responding to skin cutaneous melanomas. The third scenario pertained to a data discovery request performed on a larger set of melanoma related biomarkers. However, unlike the other two scenarios, this scenario concerned a data discovery request from non-SPHN affiliated commercial research institute, for conducting drug discovery on an anti-melanoma drug. For each scenario, participants were asked whether they would accept the request with general consent from patients, request specific informed consent, or refer the matter to an ethics committee. In the alternative, patients were given the option to indicate another method of handling the request. For each scenario, participants were asked an alternative question as to whether they would permit the feasibility request if general consent had not been sought from patients. The Swiss Academy of Medical Sciences general consent form was used as the template for all the vignettes in our interview guide.

Part B of our vignette contained two scenarios. The fourth scenario concerned a data erasure request for patient data from the second scenario of Part A that was included as part of a cohort. In this scenario, the patient was concerned about an adverse finding regarding their visa status [39]. The fifth scenario was identical to the fourth scenario, except that the patient’s data had already been included in a cohort that was sufficiently large for research and sent for publication. Both the background and scenario mentioned that personally identifying data was not stored as part of the ledger. Instead, the locally available data was linked to the data via by a cryptographic hash which linked to the record in the local ledger. This hash could be deleted, breaking the link between the data and the ledger, and making the patient’s data unavailable for further research.

**Fig. 1:**
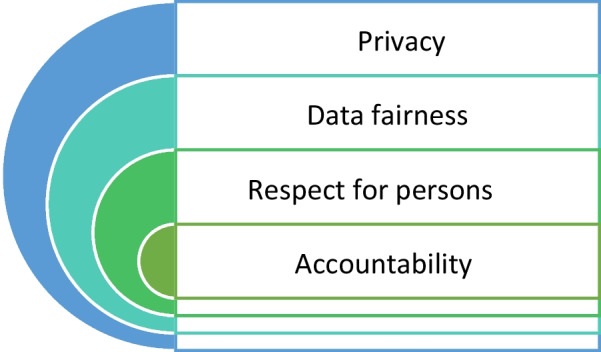
The four ethical processing factors from the SPHN Ethical Framework for Responsible Data Processing in Personalized Health

In addition, for each of our scenarios we coupled the qualitative answers with a set of Likert-like scale questions for the four ethical processing factors from the SPHN Ethical Framework for Responsible Data Processing in Personalized Health [31]. These four ethical factors were privacy, data fairness, respect for persons, and accountability (see Fig. 1). The purpose of this was to assess the degree to which interviewees rated the importance of competing ethical factors in research. The vignettes and questionnaires used in this project are contained in Additional file 1.

An initial list of interviewees was compiled by examining the web pages of relevant institutions in Switzerland to see whether there were any appropriate subject matter experts. We targeted our survey at clinical data management experts, data protection experts, clinical ethics advisors, in house legal counsel, external legal advisors affiliated with institutions and health policy experts. These interviewees work at institutions across the different linguistic and geographic barriers in Switzerland. Specifically, these included the five major university hospitals,^1^ universities and research institutions. We also drew interviewees from any institutions involved in ethics or legal governance for healthcare. Interview invitations were sent out to those participants who had a publicly available email address. After two weeks, reminders were sent out to interviewees. If interviewees had another person that they thought was a more appropriate fit for the research project, we asked them to nominate another set of potential interviewees.

Ethics approval was obtained for this project with the ETH Zürich ethics review committee (2019-N-69). Interviewees who agreed to participate in our research project were first asked to sign a consent form and were then asked again to provide verbal consent at the start of the interview. We also sought approval to perform chain sampling for this project so at the end of each interview we asked our interviewee groups for recommendations on other interviewees. Some interviews were conducted with more than one interviewee as part of a group. We obliged this request on the grounds that it would grant results more consistency if multiple experts from the same institution agreed on answers. Five potential interviewees opted out of this study because of concerns that they did not have expertise to participate in the study. All interviews were recorded with two devices, one device provided by the ETH Zürich Audio Visual department, and one device owned by the first author. The interviews were conducted either in person or via Voice Over IP (VoIP) software such as Skype or Zoom. On average, the interviews took approximately 42 min each to complete.

The interviews were transcribed and then inductively-deductively coded for themes by the first author. This inductive coding process involved the first author reading through the transcript and familiarising themselves with the answers of the interviewee and identifying the interview’s justifications. For example, if the interviewee wanted an ethics committee to review a data discovery request due to existing policy, this answer was labelled as ‘policy consistency’. The first author then generated initial codes around the interview questions and answers. These included whether the interviewee would release the data without further consent, seek informed consent from patients or refer the matter to an ethics committee. These initial codes were then used to generate further codes from the research, such as reasons for an interviewee’s decision or the interviewee’s understanding of technical terms. For example, these could include why the interviewee would refer the matter to an ethics committee for examination or the meaning of anonymised data [40]. Once these initial codes were applied, the first named author grouped them into themes for each vignette. This grouping aided the analysis of the results depending on the scenario and the circumstances [41]. This qualitative data analysis was complemented with numerical data analysis to measure how many interviewees were willing to accept data transfer requests under the scenarios depicted in our vignettes.

## Results

Overall, 13 interview sessions with 16 interviewees were conducted between September 2019 and September 2020. Data collection was extended due to a lack of immediate uptake from participants (in part due to the COVID-19 pandemic), and to achieve theoretical saturation [42]. However, previous research has indicated that as few as 12 interviews are sufficient to achieve thematic saturation [43]. Accordingly, the interviewees included in this study were sufficient to achieve theoretical saturation.

Demographic details for each of the interviewees are included below in Table 1:

**Table 1:** Demographic characteristics of participants

Characteristic	Categories	N in Sample
Gender	Male	8
	Female	8
	Other	0
Occupation	Lawyer	6
	Health Policy Researcher	2
	Data Governance	4
	Ethicist	1
	Scientific Researcher	3
Education	MS or equivalent	3
	LLM or equivalent	5
	PhD or equivalent	8
Affiliated Institution	University Hospital	10
	Research Institute	4
	Policy Centre	2
Total		16

### Part A

#### First scenario—Data discovery on non-genetic health data

For the first scenario (demonstrated by Additional file 1: Figure S1), interviewees groups were prepared to accept the request if general consent had been obtained from the patients (n = 11) or would refer the matter to an ethics committee for further approval (n = 5). However, the reason for approving the request changed between the different groups that were interviewed. Four interviewee groups pointed highlighted that the data being requested in the circumstances was aggregated data. Because this data was aggregated data, and not personal data, it therefore could be shared without the need for further informed consent. However, the interviewee group mentioned above also highlighted they would inspect the request to see what types of data were being shared before permitting the request. This answer was justified on the grounds that there was an orthogonal risk of reidentification from repeated queries. Another interviewee who was prepared to refer the request to an ethics committee highlighted how in-patient/out-patient dates could be used to single out one or more records. One interviewee who said that they would accept the request without informed consent mentioned that they wanted to ensure that, from a technical perspective, only records where general consent had been sought were available.

#### Second scenario—Data discovery on genetic health related data

For the second scenario (demonstrated by Additional file 1: Figure S2), some interviewees did not distinguish between genetic and non-genetic health related data. Therefore, they would have still agreed to make this data accessible if general consent had been obtained from the patients. The most common justification for this reasoning was that general consent would permit both access to anonymised genetic and non-genetic health related data. This conclusion is consistent with Articles 32 and 33 of the Human Research Act. Article 32, Paragraph 2 provides that further use can be made of genetic data if informed consent has been sought from the patient beforehand. Likewise, Article 33, Paragraph 2 permits further non-genetic data if informed consent has been sought from the patient beforehand. Several interviewee groups (n = 6) expressed doubt as to whether genetic data could be ever considered anonymised data due to the high potential for reidentification. However, two interviewee groups were prepared permit access to genetic data with general consent only. Their justification for this decision was that the mutation in the scenario was relatively common and so therefore could not be used to identify individuals. Conversely, another interviewee who would have permitted the request on non-genetic data with general consent would not have permitted access to this data without specific consent from the patient. We will return to this comment when addressing the legal and ethical considerations from this paper.

#### Third scenario—Data discovery on genetic health related data by a commercial research institute

For the third scenario (demonstrated by Additional file 1: Figure S3), all interviewees were willing to provide access to a commercial institute, provided this organisation agreed to be bound the SPHN principles. The same concern regarding checking the types of genetic data requested and the potential for reidentification remained for this scenario. Further, interviewees disagreed on how the relationship between the data custodian and the requesting commercial should best be handled. On one hand, four interviewees highlighted how, in their experience patients, were willing to support or even participate in research. Despite this willingness, these interviewees argued that if patients were not made aware that their data might be used for commercial research, it was questionable whether informed consent had been obtained in the circumstances. On the other hand, two other interviewee groups highlighted that it was the legal responsibility of the institute sharing the data to ensure compliance with data protection and research ethics laws. Accordingly, these interviewees argued that they would ensure the requesting institution had signed appropriate data transfer and use agreements before sending the data.

In a similar fashion, another interviewee mentioned that for this scenario they would refer the request to an ethics committee to ensure that the appropriate contractual mechanisms were in place to transfer data. Further, this interviewee mentioned that pharmaceutical companies often contacted their institution to run feasibility studies and determine whether there were enough patients. Finally, several interviewee groups mentioned the reputability of the requesting institution, as well as where they were located (n = 5), as a factor in permitting feasibility requests. This decision was justified on the grounds that Switzerland has assessed several jurisdictions (such as EU member states) as offering adequate data protection laws. In these cases, these interviewees would be prepared to send data to institutions or private companies in these jurisdictions. However, for other jurisdictions which did not offer adequacy status with EU laws, our interviewees explained they would exercise greater caution in permitting access.

A consistent theme that emerged across all three scenarios amongst interviewees was guaranteeing that the requesting researcher or institution needed the data for specified, explicit and legitimate purposes. Irrespective of the type of data, interviewees mentioned that it was important to guarantee that the data requested matched the purpose stated by the requesting institute. This perspective is consistent with Art 5 of the GDPR, which postulates that personal data shall be collected for specified, explicit and legitimate purposes and not further processed in a manner that is incompatible with those initial purposes. This principle is known as ‘purpose limitation’ [5]. The practical effect of the purpose limitation is that data must be collected for a particular research or statistical processing purpose with the explicit consent of research subjects [44]. In the alternative, there may be limited circumstances where data can be collected for research purposes without explicit and informed consent. These alternatives are discussed in further detail in the discussion section.

Another repeating theme that emerged across all scenarios was the question of general consent and appropriate information to be included in this form. Several interviewees raised concerns about the form that was supplied during the interview sessions. These interviewees felt that patients should be given the option to opt out of having their encoded and anonymised data used for research purposes. If patients were only allowed to opt out or refuse to consent to having their encoded data, but not their anonymised data used for research purposes, these interviewees reasoned that this would impinge on patient rights. One interviewee voiced concern about whether patients would have the capacity to understand the technology used to access and store patient data. According to this interviewee, patients might feel a lack of trust in the DPPH platform if they believed it was used to access their data and bypass the oversight of a research ethics committee. One point of concern was the proliferation of institution specific general consent forms (n = 8).

Several interviewees mentioned that they would be uncertain about reusing data from another institution if that institution used a different type of informed consent form. Further, one interviewee mentioned that general consent forms had only been introduced into their hospital recently. As a result, the number of patient records where general consent had been sought available for research was relatively small. The lack of a uniform general or prospective consent form is frequently reported as an issue in both Switzerland and other jurisdictions. However, several interviewees noted that a potential solution to these problems existed via Article 34 of the Human Research Act. This provision permits data without explicit consent with ethics committee approval if it would be impossible to obtain consent, no refusal exists, and the research goals outweigh the rights of individuals. We will discuss strategies on how to deal with the proliferation and variety of consent forms in the discussion below.

Another point of concern related to expertise with respect to the scenarios. As mentioned previously, 5 potential interviewees who were contacted to be interviewed refused to participate on the grounds they lacked technical expertise to answer the questions. This concern reflects a broader issue where decision making institutions, such as institutional review boards, might lack the educational and practical background to adequately assess the risks of computational science research. This lack of experience is by no means an individual failing and is an artefact of the lack of experienced researchers available to ethics committees to provide this expertise [45].

**Fig. 2:**
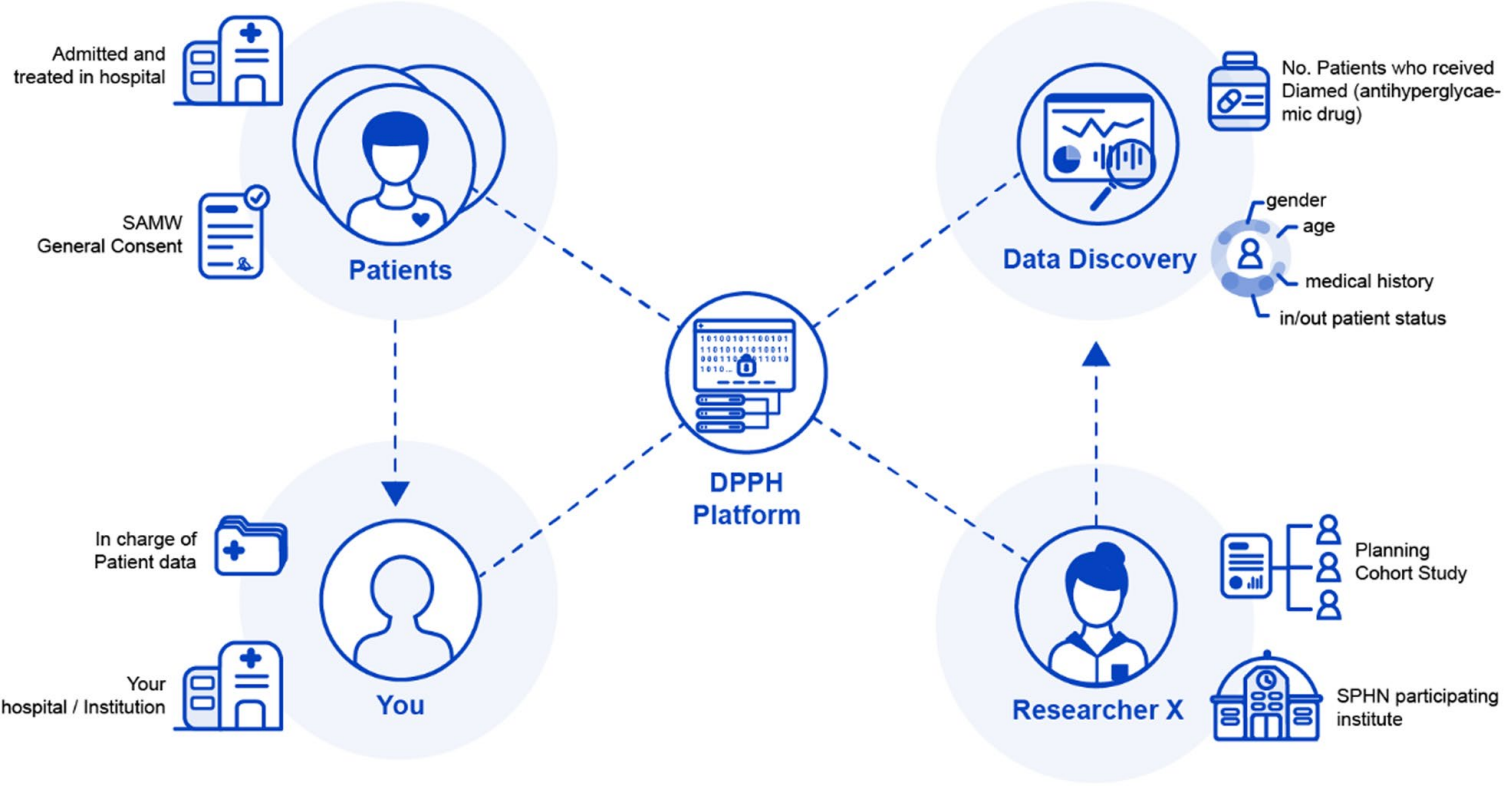
Relationship between the different actors for the DPPH platform

Figure 2 summarises the relationship between the different actors who are involved in data discovery requests on the DPPH platform.

### Part B

#### Scenario four—Erasure request before publication

For the fourth scenario (demonstrated by Additional file 1: Figure S4), a consistent theme was the competing ethical considerations at play. On the one hand, most of our interviewees at universities and research institutes explained that it was important to respect the wishes of patients and guarantee their privacy. In particular, the scenarios concerned a patient wishing to delete their data because of the validity of their residency permit. In addition, under Article 5 of the FADP controllers are obliged to delete any information that is incorrect or incomplete about a data subject, granting a limited right of erasure [46]. On the other hand, interviewees for hospitals noted they had legal and institutional responsibilities to guarantee the completeness of data. These legal obligations include being able to conduct audits on patient data, as well as cantonal legislative requirements not to erase any patient data. Finally, some interviewees mentioned that an erasure request would be a relatively rare occurrence. This observation reflects similar perspectives regarding erasure requests under the GDPR [47].

From an ethical perspective, the need to make sure that data used in research was openly available was also an important consideration. This dilemma highlights some of the ethical conflicts associated with deleting patient data. The interviewees who were not prepared to delete local dated noted the concern of the patient in this scenario did not relate to the erasure of data for treatment purposes. Maintaining patient data is important for ensuring ongoing quality of care and auditing. Further, deleting research data raises ethical considerations regarding wasting data and the time of research participants [48]. However, emergent models of patient data ownership are increasingly challenging the idea that healthcare professionals rather than patients control data [49]. For this research project, interviewees also highlighted that under the current Federal Act on Data Protection, data can only be processed for a scientific publication unless it has been anonymised. Therefore, these interviewees were prepared for aggregate (and therefore anonymised) data to be included in a publication provided that no future research could be conducted with the patient’s data. The patient’s data would either not be deleted or sealed so that it remained accessible for clinical treatment but not for research.

#### Scenario five—Erasure request after publication

With specific regards to the fifth scenario (demonstrated by Additional file 1: Figure S5), our interviewees highlighted different sources of potential authority to justify processing the patient’s request. One interviewee pointed out that the general consent form in the scenario guaranteed that patient data would not be available for new research projects if the patient withdrew their consent. According to this interviewee, this definition would be wide enough to encapsulate Another interviewee highlighted the fact that Article 10 of the Human Research Ordinance requires data to be anonymised once it has been evaluated. Further, the previously mentioned requirement to anonymise data before it is included in a scientific publication meant that these interviewees believed no personally identifying data could be lawfully included as part of a publication. Nevertheless, one of these interviewees conceded that from an ethical perspective, determining whether to delete data would depend on the size of the study. For example, a study with ten patients might be significantly more impacted by an erasure request than a study with thousands of patients. Therefore, this interviewee explained that they would attempt to use a “technical consensus” in determining whether to erase data.

#### Likert scores

The aggregate values for the Likert scores are displayed in Table 2. It should be noted that one interviewee group were not prepared to offer scores for the scenarios.

**Table 2:** Aggregate values for each of the four SPHN ethical processing principles for the five scenarios

Scenario	Privacy	Data fairness	Accountability	Respect for persons
Scenario 1 (data discovery on non-genetic data by a public institution)	6.25	5.67	5.67	5.50
Scenario 2 (data discovery on genetic data by a public institution)	6.75	5.25	5.75	6.50
Scenario 3 (data discovery on genetic data by a private institution)	6.91	5.33	6.17	6.67
Scenario 4 (erasure request before research)	6.42	3.83	5.75	6.50
Scenario 5 (erasure request after research)	6.58	4.42	5.92	6.50

## Discussion

To aid with the discussion, we will split this section into legal and ethical issues.

### Legal issues

In practice the distinction between genetic and non-genetic data under the Human Research Act was not reflected in the answers given by interviewees. Instead, interviewees adopted a more contextual approach for determining when genetic or non-genetic data would be sensitive personal data. This approach confirms what the authors have written in previous studies regarding orthogonal risks to privacy from processing aggregate data [1, 2]. These orthogonal risks include circumstances where an attacker possesses other information that can be used to identify individual records or conduct inference attacks on aggregated data. These risks can be accentuated when dealing with genomic data, which can be used to identify individuals even more precisely [50]. Likewise, with big data and machine learning techniques proliferating in social sciences research, it can be difficult to determine whether research protocols fall within the scope of ethics committee purview [51]. Accordingly, data encrypted using advanced privacy technologies such as HE will not be anonymised where the entity holding that data possesses a method to decrypt it. This approach is analogous to the treatment of pseudonymised data and encryption keys under the GDPR [7]. When handed to a third party without the means to decrypt this data, the data will be anonymised data. However, depending on the data that has been released, there may be an orthogonal risk of singling out one or more records. Further, the fact that privacy and respect for persons were the most highly rated scores on our Likert scale indicates the importance of guaranteeing participant privacy to interviewees.

There are several strategies that could be used separately or in concert to resolve this problem and reduce the risk of reidentification. The first is to combine data discovery requests (and accompanying privacy enhancing technologies) with role-based access control. This would allow data custodians to certify the requesting clinician, researcher, or institution to determine that they had been approved access. Role based access control could also be used to prevent repeated requests that might be used to reidentify an individual. The second would be to adopt a more contextual approach for determining when data was encoded or anonymised beyond the distinction between genetic and non-genetic data under Swiss legislation [49]. For example, one group of interviewees mentioned that whole genome sequencing data would carry significantly less risks than germline or aggregate results about the number of single mutations. Another interviewee also mentioned that certain types of non-genetic data, such as inpatient and outpatient status, could be used to reidentify patients. This contextual based approach could be combined with role-based access control to decrease the risk of patients being reidentified. Likewise, as interviewees suggested, patients could be given more control to prevent the upload of potentially sensitive patient data. Finally, from an organisational perspective, an ethics review committee could establish a protocol for determining when the risk of reidentification is sufficient that a feasibility request is referred for ethics review. It should be noted that a mechanism for a ‘jurisdictional request’ already exists for an ethics committee to determine whether a particular project should undergo ethics approval [52]. A version of this ‘jurisdictional request’ could be made to a specialist in computer science or statistics to reassess the potential for reidentification.

Another important consideration raised by some interviewees was the status of the different entities responsible for processing. Three interviewees requested that we clarify who they were meant to be in the scenario prior to giving their answers. Their justification for this response was that the responsibilities for data custodians such as hospitals and requesting agencies such as universities and private research companies differ under data protection law. Specifically, data custodians should be treated as data controllers under the GDPR and FADP. However, the authors have previously assessed requesting institutes and companies as joint controllers who are equally responsible for compliance when using advanced privacy enhancing technologies [2]. Therefore, it is important that the contractual responsibilities of each processing entity are clarified prior to processing. One interviewee mentioned the BioMedIT Network, the output of another SPHN driver project. The purpose of BioMedIT is to create a platform for collaborative data analysis without compromising data privacy [53]. This interviewee mentioned that queries could be performed on data using the BioMedIT infrastructure. A BioMedIT Network node would be treated as a data processor under data protection law, rather than a controller, as the operators of this node are appointed to process data. However, all these details would need to be clarified in contractual terms between the entities responsible for processing data. In addition to clarifying the terms governing data processing, this contract would ensure an appropriate physical and organisational separation of encryption keys to prevent reidentification [2].

A final legal issue that needs to be clarified is the terms of general consent forms. As mentioned previously, several interviewees noted that there had been a proliferation of general consent forms. This problem is well recognised within the Swiss context, and studies have been dedicated to developing a nationwide integrated framework [54]. Therefore, interviewees were concerned that a general consent that was recognised and valid for one hospital would not be valid for another. Further, one interviewee mentioned that a general consent form should not only allow a patient to opt out of having their data encoded, but also having their data anonymised. This distinction is important; once a patient’s data is anonymised or aggregated, it cannot be traced back to them. Therefore, the patient loses the ability to exercise their rights with respect to their own data [55]. Another interviewee from a university hospital noted their institution had developed a general consent form that allowed opt outing of further use for both encoded and anonymised data. Although this consent form went beyond the legal requirements, it nevertheless offered the patient more control over their data compared to other general consent forms. Accordingly, amending existing ethics forms to offer patients more control over their data, even once it has been anonymised, could be an important strategy to guarantee social licence. This discussion dovetails into the ethical discussion of general consent forms below.

### Ethical issues

General and specific consent forms are also relevant from an ethical perspective. One interviewee, who refused to give specific scores for the Likert scales, argued that general consent forms could be used strategically by researchers. The effect of this use would be to limit the liability or the ongoing responsibility of the research team, whilst maximising reuse of the data. Likewise, this interviewee believed that patients would see advanced privacy enhancing technologies as a method for researchers to reduce their ethical responsibilities. Although privacy enhancing technologies are primarily designed to reduce the risk of data breaches, patient trust and social licence are essential to reusing patient data for research purposes [56]. Accordingly, failing to ensure that advanced privacy enhancing technologies have sufficient public licence could undermine the willingness of patients to permit their data to be processed using these technologies. Another interviewee mentioned this public trust could be accentuated with a general consent form that would allow the patient to seek further information about the research projects their data is used for. This general consent form should highlight whether a patient’s data might be used for commercial research purposes. In the alternative, other researchers have focussed on the concept of meta consent. Holm and Ploug describe a meta consent model in which patients can specify their consent, data, and projects for which this data can be used. First, patients can specify whether they grant specific consent to a particular research project, or broad consent for multiple research projects. Secondly, patients can consent to different types of data being used for research purposes (including patient records and linked data). Thirdly, patients can consent to their data being used for non-commercial and commercial purposes [57]. Ploug and Holm have subsequently presented a proof-of-concept mobile application that can be used to record consent [58]. Accordingly, a similar approach should be adopted with the use of advanced privacy enhancing technologies and distributed ledger technologies. For privacy enhancing technologies, patients should have the option to indicate whether they would be willing to let their data be used for feasibility studies. The EU has recognised the need for a uniform consent model to encourage the secondary use of data, and accordingly the European Commission proposed a new Data Governance Act in 2020 [59]. This new act is discussed in further detail in the next section, which addresses European wide strategies for secondary uses of data.

Connected to ethical considerations regarding consent are questions of both practitioner and patient education. As mentioned previously, 5 experts contacted as interviewees refused on the grounds that they lacked knowledge about advanced privacy enhancing technologies or DLT. Therefore, both researchers and decision-making bodies, such as research ethics committees, should receive ongoing training about computational technologies and data driven research. This training would help researchers and decision-making bodies develop a consistent understanding of terms such as anonymisation and balance competing ethical considerations that might spring from its use [45, 60]. Similarly, one interviewee questioned whether a patient could give explicit and informed consent to having their data processed using this technology. However, as another interviewee explained, it might be difficult to explain homomorphic encryption and DLT to a patient in a fashion that was comprehensible. Accordingly, this interviewee suggested that, in addition for participants to find further information about their research, the general consent form should include a concise summary of these technologies. Further, the first interviewee above mentioned that ongoing publication education and awareness campaigns could be used to help encourage acceptance of advanced privacy enhancing technologies. One limitation of this paper is the focus on expert interviews, a point raised by many interviewee groups. Future studies could provide vignette scenarios to patients to examine how they would respond to these requests and in what circumstances they would accept their data being uploaded. Likewise, this paper focused on interviews with legal experts, who do not necessarily have subject matter expertise on advanced privacy enhancing technologies. Future studies could replicate these questions for computer scientists, biostatisticians and data scientists handling health data. However, these questions would need to be slightly modified to provide greater context for ethical and legal concepts, given that potential interviewees may not have subject matter expertise in these fields.

### Applicability outside of Switzerland

One challenge that needs to be addressed with this project is the question of compatibility with both national and supranational legislation outside of Switzerland. Although the Human Research Act explicitly recognises the potential for general consent forms to be used for research, the lawfulness of general consent under the GDPR is unclear. Article 9(1) of the GDPR prima facie prohibits the processing of special categories of data, including health related and genetic data. However, Article 9(2)(a) overturns this prohibition if free, informed, and explicit consent is obtained from data subjects. Recital 33 of the GDPR provides that subjects should be able to give their consent to certain areas of scientific research. Implicitly, this Recital could support the need for general consent [61]. However, the former Article 29 Working Party subsequently held that Recital 33 cannot be used to dispense with the requirements for a well-defined research purpose. Instead, the goals of research can only be described in more general rather than specific terms [62]. Although not denying researchers the ability to rely on general consent under the GDPR, these guidelines significantly reduce the scope of broad consent. Nevertheless, Article 9(4) permits member states to impose further conditions on the processing of genetic and health-related data. Therefore, the boundaries for informed consent may very much depend on a case-by-case basis.

One development that may aid secondary uses of medical data across borders is the European Commission proposal for a Data Governance Act mentioned previously [59]. The purpose of this Act is to create a framework to encourage reuse of public sector data for commercial and ‘altruistic purposes’, including scientific research. The Data Governance Act does not mandate reuse of public sector data, such as data subject to intellectual property protections or highly confidential data. In this context, ‘public sector data’ includes both personal data as governed under the GDPR and non-personal data. However, Article 22 of the proposed Data Governance Act allows the European Commission to create implementing acts for a ‘European data altruism consent form’ to allow for uniform consent across the EU. This consent form must be modular so that it can be customised for different sectors and purposes. Further, data subjects must have the right to consent to and withdraw their data from being processed for specific purposes. The Data Governance Act has not yet entered into force, and the current draft could still undergo significant revisions. However, the Data Governance Act could act as a mechanism to standardise general consent between different EU member states, ameliorating the challenges with cross border transfers of data. The Data Governance Act could also act to empower data subjects so that they can exercise greater control over how their data is used for research [63].

With respect to erasure, and the GDPR’s right of erasure under Article 17, the drafters of the GDPR recommended that personal data be not stored in any blockchain ledger. If data must be stored in a DLT platform, that storage should be coupled with adequate access control mechanisms [64]. However, Article 17 paragraph 3 creates an exception for data collected for public health and safety purposes (paragraph 3(c)). In the alternative, the right to be forgotten cannot be exercised where personal data is archived for research or statistical processing, and erasure would render the purpose of research impossible (paragraph 3(d)). Although the interpretation of this exception is uncertain, it offers a relatively broad scope for researchers to continue to process data, despite erasure requests [55]. Nevertheless, it is important to not only consider the legal but also the ethical consequences of refusing erasure requests. Specifically, the decentralised ledger implementation used in MedCo allows links to locally stored data to be erased, thereby complying with GDPR erasure requests.

## Conclusions

In this paper, we described an interview study on the use of HE and DLT for processing patient data. This interview study was conducted with experts from Swiss hospitals and research institutes, and included legal and clinical data management staff, along with clinical and legal ethicists. We interviewed these stakeholders with two sets of five vignettes concerning feasibility or data discovery requests, and data erasure requests. With respect to the first set of requests, most of our interviewees were prepared to permit processing, provided that general consent had been obtained from patients to do so. Accordingly, advanced privacy enhancing technologies have the potential to fill the regulatory gaps that exist under current data protection laws in Switzerland. However, our interviewees also highlighted the importance of assessing the risk of reidentification from data released as part of a feasibility request. In addition, our interviewees identified that existing consent practices may not be sufficient to explain the technical complexity of advanced privacy enhancing technologies. Depending on the canton where interviewees were located and cantonal or institutional retention requirements, interviewees were willing to delete links to data in a distributed ledger or to that locally stored data. However, our interviewees also expressed concern regarding potential consent issues concerning technological complexity. Therefore, this study demonstrates that a holistic approach needs to be taken to introducing HE and DLT as a mechanism for patient data management. It is important to recognise that social licence and public trust from patients and physicians is as important as legal compliance. Specifically, general consent forms should be amended to offer patients the opportunity to opt out to having their data anonymised using advanced privacy enhancing technologies. Further, patients should have the opportunity to indicate what types of their data they wish to be made available for data discovery. Finally, a public education campaign should be targeted at explaining how these technologies work to give patients the opportunity to understand how their data will be processed. This education campaign will help support the social licence required for these initiatives. Future research should address how patients might respond to the use of these technologies to process their data.

## Supplementary Information

**Additional file 1**. Interview Questionnaire and Vignettes.

## Data Availability

The datasets generated during and analysed during the current study are not publicly available due to the low number of interviewees and high potential for reidentification, but are available from the author Ienca on reasonable request.

## Footnotes

1 The five main university hospitals in Switzerland are the Geneva University Hospitals (*Hôpitaux universitaires de Genève*, or HUG), the University Hospital of Bern (*Universitätsspital Bern,* or Inselspital), the Lausanne University Hospital (*Centre Hospitalier Universitaire Vaudois*) and the University Hospital of Zürich (*Universitätsspital Zürich,* or USZ).
