# Supplementary material for: Health data privacy through homomorphic encryption and distributed ledger computing: an ethical-legal qualitative expert assessment study
Source: BMC Med Ethics. 2022 Dec 1;23:121. doi: 10.1186/s12910-022-00852-2 (PMC9713155; doi:10.1186/s12910-022-00852-2)
Supplement: Supplementary file 1 — Additional file 1. Interview Questionnaire and Vignettes. [file 12910_2022_852_MOESM1_ESM.docx]

# Appendix A:

## **Interview Questionnaire**

### DPPH Data Sharing Platform: ELSI Consultation Framework

The DPPH platform is designed to encourage the exchange of biomedical and health related data for P4 (Predictive, Preventative, Personalized and Participatory) medicine. The DPPH platform relies on homomorphic encryption and differential privacy to ensure the security of patient data. The DPPH platform also uses distributed ledger technology to guarantee auditability and transparency for data exchange. Specifically, this ledger permits patients to track the transfer and usage of their data with no single point of weakness that could permit record modification. The DPPH platform uses distributed ledger technology to achieve this goal via an immutable ledger of encrypted links to locally stored patient records. These patient records are stored locally at DPPH partner institutes and hospitals. This ledger cannot be modified without consensus from the participating nodes.

The purpose of these vignettes is to assess the balance between transparency and auditability guaranteed by patient records, and the right of privacy and data protection for patients. The first three vignettes below describe hypothetical scenarios where data discovery requests have been made against patient data stored at your institute. Specifically, they involve the external researcher determining whether there is a sufficient study cohort to conduct their research. For each scenario, you will be asked what queries you would permit, as well as any ethical or legal concerns you would have with respect to the queries. The fourth vignette describes a scenario where a patient has requested their data be deleted from the DPPH platform. For this scenario, you will be asked what form of erasure request you would permit, and what ethical or legal concerns you would have respect to the request.

In your answers, you may refer to any applicable or relevant ethical frameworks or legal instruments. These instruments include those referred to in the Glossary below, as well as any other instruments that you regard as relevant.

### Glossary

Anonymised data: Anonymised data refers to personal data where all personal identifiers have been removed from the data, and reidentification is impossible or requires undue effort. Reidentification of data can occur through either direct identification or indirect identification.

Data discovery: Data discovery refers to a process in which data from disparate sources are collected together into a single repository to determine whether it is sufficient for research.

Encoded data: Encoded data refers to personal data where all identifiers have been replaced with a code. The data cannot be identified without reference to this code. Under EU data protection legislation (such as the *General Data Protection Regulation*), encoded data is referred to as pseudonymised data.

Ethical frameworks: non-legislative frameworks for the conduct of medical and biotechnology research. These instruments may include the SPHN Framework for Ethical Conduct of Research, or the SAMW General Consent Form.

Legal instruments: legislative frameworks that apply to the conduct of medical and biotechnology research. These instruments may include the Federal Act on Research Involving Human Beings (the HRA) and the Federal Act on Data Protection (FDAP). These instruments may also include European Union legislation (such as the GDPR), Council of Europe legislation (Convention 108) or US legislation (HIPAA).

### Part A: Query Requests

Scenario 1: Data Query on Non-Genetic Health Related Personal Data from SPHN participating institute

Aim: *To conduct analysis of patients with diabetes after receiving drug related treatment.*

Description: *Researcher X, who works at a separate SPHN participating institute, wishes to perform data discovery via the DPPH platform on data your institute holds about your patients. Researcher X wishes to perform data discovery to determine whether there are enough patients who received Diamed, an antihyperglycaemic drug, to construct a study cohort. This data includes age, sex, past medical history, inpatient or outpatient status. Researcher X also wishes to collect data on whether patients received Diamed or not. General consent was collected from patients for the use of their data (using the SAMW General Consent form) when they were admitted to hospital.*

*Stage 1:*

For the data requested by Researcher X, indicate what *data discovery* queries you would permit, assuming that no other processing occurs (you may indicate more than one option below).

1. Accept the data discovery request on patient non-genetic and outcome data without a request for informed consent;
2. Require informed consent for data discovery on patient genetic and outcome data;
3. Refer the request for data discovery on patient genetic and outcome data to your institution’s research ethics committee; or,
4. Other (*please specify*)

For the data discovery queries you would permit, please justify your reasons. For the data discovery queries you would *not* permit, please justify your reasons.

*Stage 2:*

Would any of your answers to Question 1 change if *general consent* had **not** been obtained from patients prior to diagnosis or treatment? Please justify your reasons.

*Stage 3:*

How important would you consider the following ethical principles with respect to the data query (on a scale of 1 to 7, where 1 is not important and 7 is very important)?

1. Privacy (confidentiality of data, access controls)
2. Data Fairness (*data availability, data sharing*)
3. Accountability (*transparency, lawfulness, auditability*)
4. Respect for Persons (*respect for dignity, basic rights, informational self-determination*)

For each of your ratings, please justify your reasons.

Scenario 2: Data Query on Genetic Data and Health Related Personal Data from SPHN participating institute

Aim: *To quantify proportion of population possessing mutation associated with skin cutaneous melanoma.*

*Description: Researcher Y who works at another SPHN affiliated institute, wishes to perform data discovery on data your institute holds about your patients. The aim of this project is to discovery a patient population with biomarkers associated with skin cutaneous melanoma. In particular, Researcher Y wishes to perform data discovery to discover patients with a mutation in the BRAF gene at position V600E or V600K. Researcher Y also wishes to perform data discovery on age, sex, inpatient or outpatient status, and patient outcome data. General consent was collected from patients using the SAMW General Consent form at the time that they were admitted.*

*Stage 1:*

For the data requested by Researcher Y, indicate what data discovery queries you would permit for *anonymised data* (you make select more than one option from those below).

1. Accept the data discovery request on patient genetic and outcome data without a request for informed consent;
2. Require informed consent for data discovery on patient genetic and outcome data;
3. Refer the request for data discovery on patient genetic and outcome data to your institution’s research ethics committee; or,
4. Other (*please specify*)

For the data discovery queries you would permit, please justify your reasons. For the data discovery queries you would *not* permit, please justify your reasons.

*Stage 2:*

For the data requested by Researcher Y, indicate what data discovery queries you would permit for *encoded data* (you make select more than one option from those below).

1. Accept the data discovery request on patient genetic and outcome data without a request for informed consent;
2. Require informed consent for data discovery on patient genetic and outcome data;
3. Refer the request for data discovery on patient genetic and outcome data to your institution’s research ethics committee; or,
4. Other (*please specify*)

For the data discovery queries you would permit, please justify your reasons. For the data discovery queries you would *not* permit, please justify your reasons.

*Stage 3:*

Would any of your answers to Stage 1 or Stage 2 change if *general consent* had **not** been obtained from patients prior to diagnosis or treatment? Please justify your reasons.

*Stage 4:*

How important would you consider the following ethical principles with respect to the data query (on a scale of 1 to 7, where 1 is not important and 7 is very important)?

1. Privacy (*security controls, protecting patient privacy, preventing unauthorized access*)
2. Data Fairness (*data availability, data sharing*)
3. Accountability (*transparency, lawfulness, auditability*)
4. Respect for Persons (*respect for dignity, basic rights, informational self-determination*)

Scenario 3: Data Query on Genetic Data from non-partner SPHN institute

Aim: *To quantify proportion of population possessing mutation associated with skin cutaneous melanoma.*

*Researcher Z, who works at a non-SPHN affiliated, private research organisation, wishes to perform the same data discovery as researcher Y. Researcher Z wishes to identify patients that have skin cutaneous melanomas, a mutation in the BRAF gene, and a mutation in the PTEN, CDKN2A, MAP2K1 or MAP2K2 genes). In particular, Researcher Z’s research is based on the fact that patients treated with vemurafenib develop resistance through mutations that activate the MAP kinase pathways. Identifying patients with this mutation may be extremely useful for the development of alternative drugs for the treatment of melanomas. General consent was collected from patients using the SAMW General Consent form at the time that they were admitted.*

*Stage 1:*

For the data requested by Researcher Z, indicate what data discovery queries you would permit on *anonymised patient data* (you make select more than one option from those below).

1. Accept the data discovery request on patient genetic and outcome data without a request for informed consent;
2. Require informed consent for data discovery on patient genetic and outcome data;
3. Refer the request for data discovery on patient genetic and outcome data to your institution’s research ethics committee; or,
4. Other (*please specify*)

For the data discovery queries you would permit, please justify your reasons. For the data discovery queries you would *not* permit, please justify your reasons.

*Stage 2:*

For the data requested by Researcher Z, indicate what data discovery queries you would permit on *encoded patient data* (you make select more than one option from those below).

1. Accept the data discovery request on patient genetic and outcome data without a request for informed consent;
2. Require informed consent for data discovery on patient genetic and outcome data;
3. Refer the request for data discovery on patient genetic and outcome data to your institution’s research ethics committee; or,
4. Other (*please specify*)

For the data discovery queries you would permit, please justify your reasons. For the data discovery queries you would *not* permit, please justify your reasons.

*Stage 3:*

Would any of your answers to Stages 1 or 2 change if *general consent* had **not** been obtained from patients prior to diagnosis or treatment? Please justify your reasons.

*Stage 4:*

How important would you consider each of the following ethical principles with respect to the data queries (on a scale of 1 to 7, where 1 is not important and 7 is very important)?

1. Privacy (*security controls, protecting patient privacy, preventing unauthorized access*)
2. Data Fairness (*data availability, data sharing*)
3. Accountability (*transparency, lawfulness, auditability*)
4. Respect for Persons (*respect for dignity, basic rights, informational self-determination*)

For each of your ratings, please justify your reasons.

### Part B: Request for Deletion

Scenario 4: Request to Delete Health Related Personal Data

*Patient N underwent Diamed treatment for diabetes at your institution two years ago, with consent for treatment and data collection being obtained using the SAMW General Consent form. Patient N has managed to control her condition and she has continued with her life without further complications. Patient N has contacted your institution to discuss her treatment. Patient N reveals that at the time she was involved in treatment, she did not have legal residency in Switzerland. Patient N has since obtained the correct working visa. However, Patient N is extremely concerned that if her data is available for inspection upon the DPPH platform, someone may discover her occupation. Patient N is also deeply concerned that if the authorities discover that she was working in Switzerland without a visa, she may be identified and criminally prosecuted. She has therefore requested your institution delete her data.*

*Patient N’s data was included in a cohort that was sufficiently large for the intended research. This data has been analysed but the findings have not yet been published.*

*Stage 1:*

With respect to Patient N’s request, would you:

1. Request that all of Patient N’s treatment records be deleted from local storage;
2. Request that links to Patient N’s database be removed from the DPPH platform, so that it is stored but not used or returned in any query; or,
3. Other (*please specify*)?

Please justify your reasons for permitting the requests described above.

*Stage 2:*

How important would you consider each of the following ethical principles associated with Patient N’s request (on a scale of 1 to 7, where 1 is not important and 7 is very important)?

1. Privacy (*security controls, protecting patient privacy, preventing unauthorized access*)
2. Data Fairness (*data availability, data sharing*)
3. Accountability (*transparency, lawfulness, auditability*)
4. Respect for Persons (*respect for dignity, basic rights, informational self-determination*)

Scenario 5: Request to Delete Health Related Personal Data

*Patient N underwent Diamed treatment for diabetes at your institution two years ago, with consent for treatment and data collection being obtained using the SAMW General Consent form. Patient N has managed to control her condition and she has continued with her life without further complications. Patient N has contacted your institution to discuss her treatment. Patient N reveals that at the time she was involved in treatment, she did not have legal residency in Switzerland. Patient N has since obtained the correct working visa. However, Patient N is extremely concerned that if her data is available for inspection upon the DPPH platform, someone may discover her occupation. Patient N is also deeply concerned that if the authorities discover that she was working in Switzerland without a visa, she may be identified and criminally prosecuted. She has therefore requested your institution delete her data.*

*Patient N’s data was included in a cohort that was sufficiently large for the intended research. The findings have been analysed and sent for publication.*

*Stage 1:*

With respect to Patient N’s request, would you:

1. Request that all of Patient N’s treatment records be deleted from local storage;
2. Request that links to Patient N’s database be removed from the DPPH platform, so that it is stored but not used or returned in any query; or,
3. Other (*please specify*)?

Please justify your reasons for permitting the requests described above.

*Stage 2:*

How important would you consider each of the following ethical principles associated with Patient N’s request (on a scale of 1 to 7, where 1 is not important and 7 is very important)?

1. Privacy (*security controls, protecting patient privacy, preventing unauthorized access*)
2. Data Fairness (*data availability, data sharing*)
3. Accountability (*transparency, lawfulness, auditability*)
4. Respect for Persons (respect for dignity, basic rights, informational self-determination)

## **Vignette Scenarios as Diagrams**


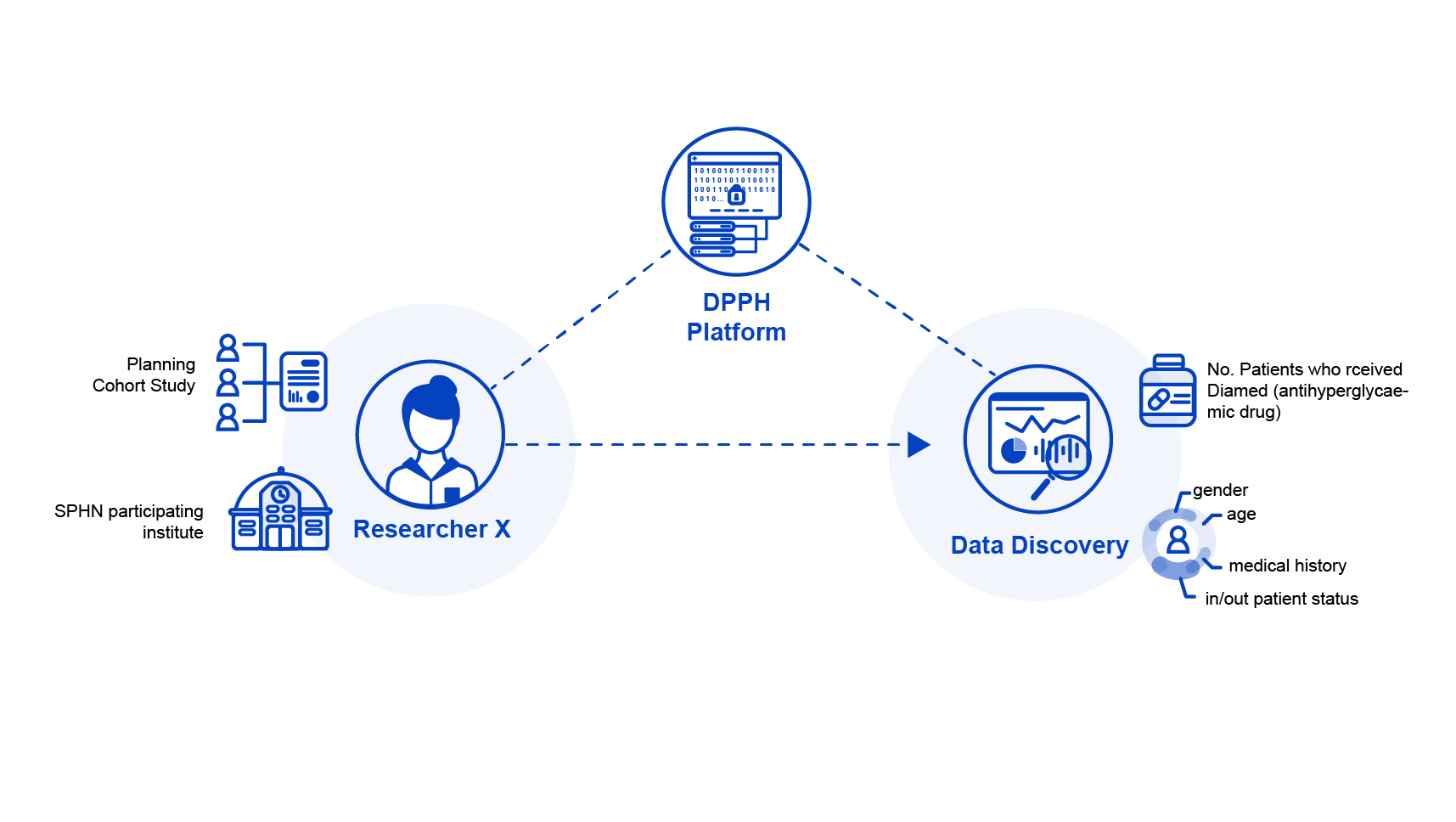


**Supplementary Figure 1:** Data Discovery Request on Non-Genetic Data


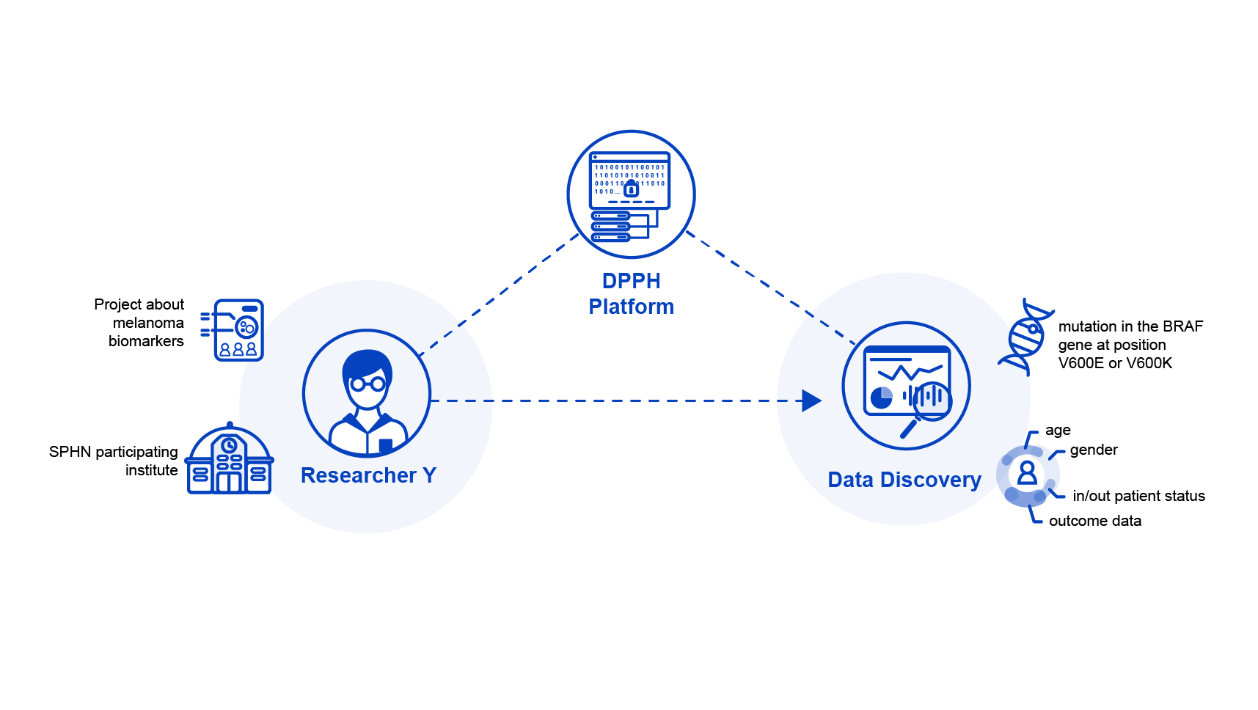


**Supplementary Figure 2:** Data Discovery request on Genetic Data from SPHN institute


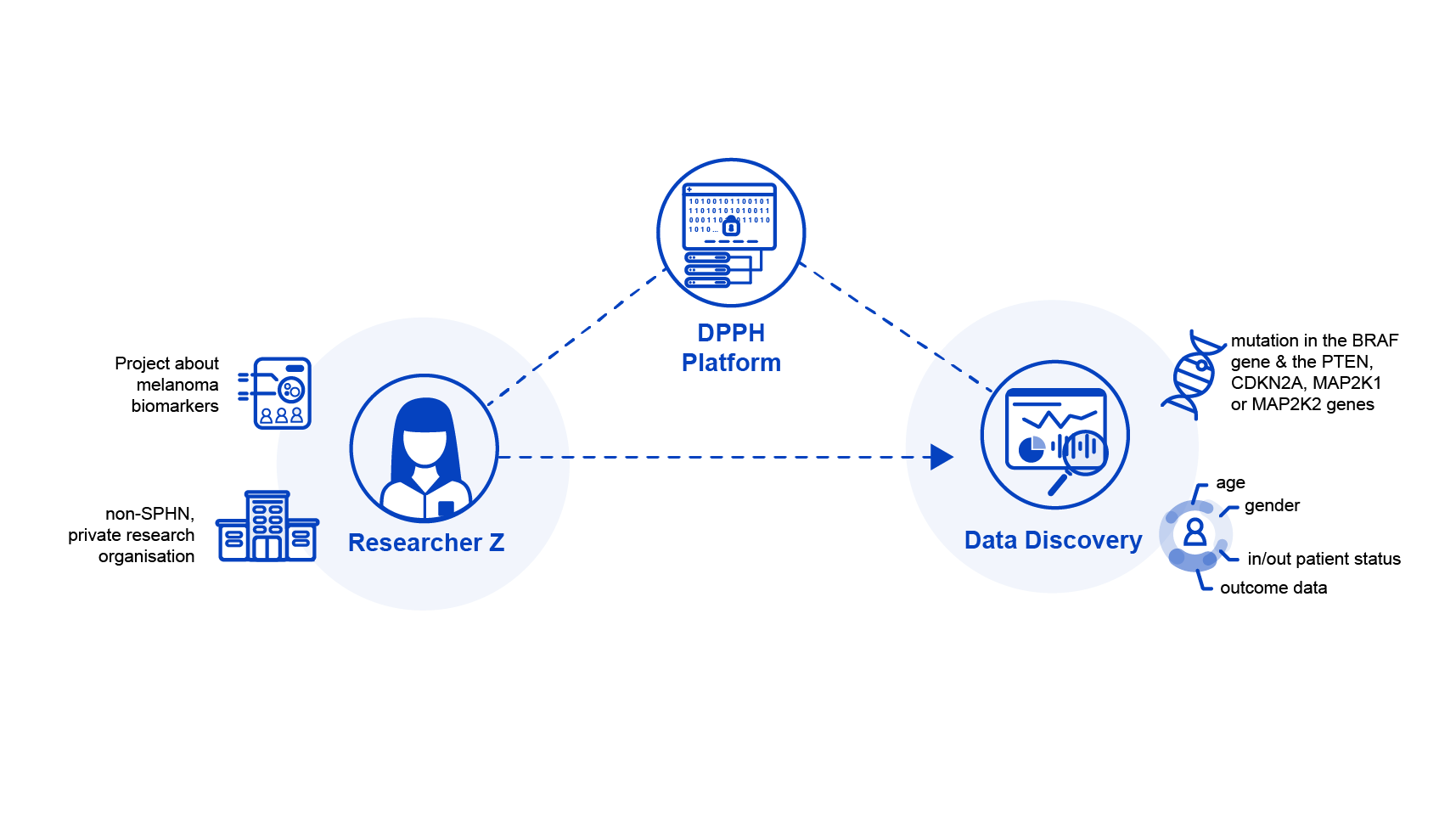


**Supplementary Figure 3:** Data Discovery request on Genetic Data from non-SPHN institute


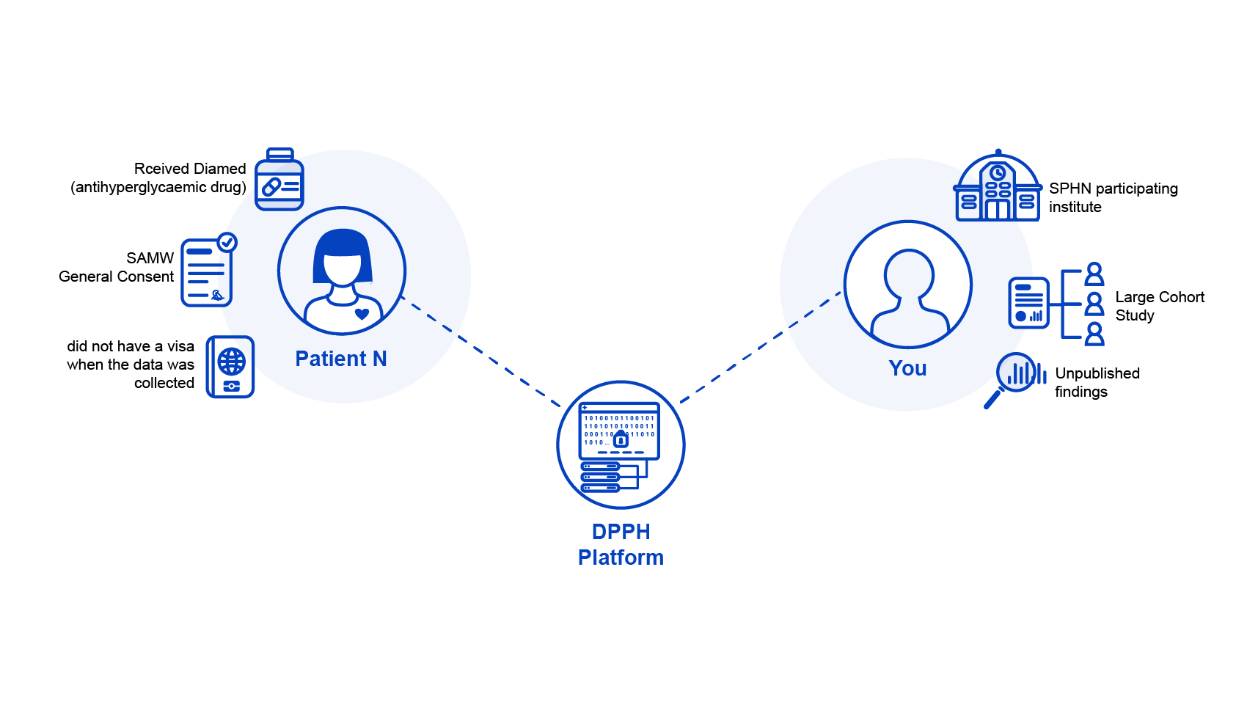


**Supplementary Figure 4:** Data erasure request to Genetic Data (no publication)


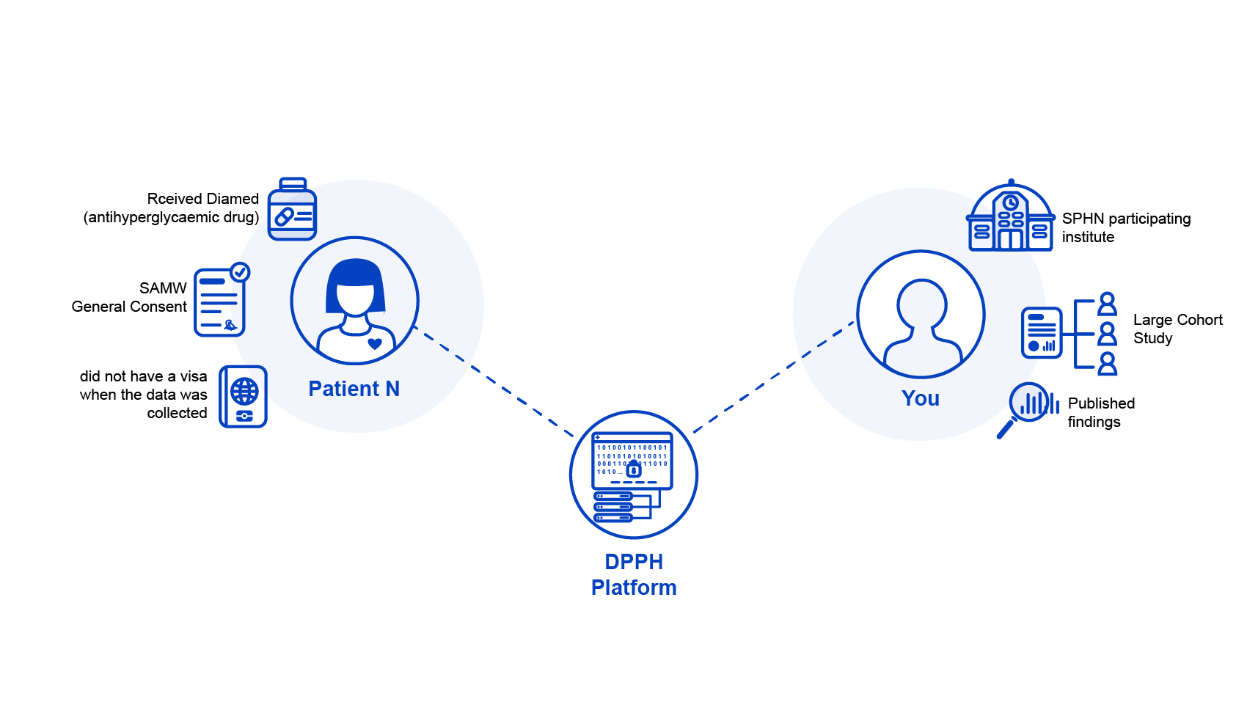


**Supplementary Figure 5:** Data erasure request to Genetic Data included in a publication
